# Characterization of Acid- and Pepsin-Soluble Collagen Extracted from the Skin of Purple-Spotted Bigeye Snapper

**DOI:** 10.3390/gels8100665

**Published:** 2022-10-17

**Authors:** Siti Nur Hazwani Oslan, Rossita Shapawi, Ruzaidi Azli Mohd Mokhtar, Wan Norhana Md. Noordin, Nurul Huda

**Affiliations:** 1 Faculty of Food Science and Nutrition, Universiti Malaysia Sabah, Jalan UMS, Kota Kinabalu 88400, Malaysia; 2 Borneo Marine Research Institute, Universiti Malaysia Sabah, Jalan UMS, Kota Kinabalu 88400, Malaysia; 3 Biotechnology Research Institute, Universiti Malaysia Sabah, Jalan UMS, Kota Kinabalu 88400, Malaysia; 4 Fisheries Research Institute, Batu Maung 11960, Malaysia

**Keywords:** extraction, fish processing waste, skin purple-spotted bigeye snapper (*Priacanthus tayenus*), collagen, characterization

## Abstract

Fish processing waste is a prospective source of collagen and a cost-effective environmental pollutant. The skin of the purple-spotted bigeye snapper (*Priacanthus tayenus*) was extracted utilising various acid soluble collagens (ASC) including acetic acid (AAC), lactic acid (LAC), citric acid (CAC) and pepsin soluble collagens (PSC). In this study, PSC (6.65%) had the highest collagen yield, followed by AAC (5.79%), CAC (4.15%), and LAC (3.19%). The maximum temperatures (*T*_max_) denaturation of AAC, LAC, CAC, and PSC were 31.4, 31.7, 31.5, and 33.2 °C, respectively. UV-VIS absorption spectra showed all extracted collagens had a range of absorbance at 230 nm, due to the presence of glycine, proline, hydroxyproline, and triple-helical collagen. Additionally, they exhibited amide A, B, amide I, II, and III peaks. SDS–PAGE identified all extracted collagens as type I. The PSC had a significantly higher (*p* < 0.05) hydroxyproline content than acidic extraction 66.3 ± 1.03 (mg/g sample). Furthermore, all samples were extremely soluble in acetic conditions at pH 5, and all collagen was soluble in NaCl up to 3% (*w*/*v*). Therefore, PSC was the best treatment since it did not impact collagen triple helical and acetic acid yielded the most collagen in ASC extraction. Overall, the analysis revealed that fish skin waste might be used as an alternate source of collagen in diverse applications, particularly in food applications.

## 1. Introduction

Collagen has become an element of food, cosmetics, biomedical products, and pharmaceuticals [1,2]. Furthermore, collagen was discovered to be the principal structural protein that supported the skin and internal organs of animals and fish [3,4]. Fish production is a promising sector in industry, with more than 196 million tons of fish expected to be processed in 2025 [5]. According to Azra et al. [6], Malaysia’s Fisheries Gross Domestic Product (GDP) has a trade value of USD 1.75 billion. The Purple-spotted bigeye snapper (*Priacanthus tayenus*) is commonly available in Malaysia, is inexpensive, and is effective for surimi production [7]. Consequently, the efficient utilisation of fisheries by-products can provide value-added products and prevent pollution [8]. To fix the situation, effective methods are needed to transform fish by-product wastes into important nutrients. A large percentage of by-products account for 50% to 70% of the original raw material, which contains collagen-rich heads, viscera, skin, bone, scales, and fins, depending on the processing method [9,10]. Moreover, fish skin has a high protein content, and collagens generated from fish skin have a wide range of applications as functional food components [2]. Furthermore, fish by-products also include a high amount of crude protein, ranging from 8 to 35%, and can be used as a source of collagen, gelatin, polyunsaturated fatty acids, enzymes, and essential amino acids [10]. Fish collagen has been used in the development of protein nutritional multivitamins, transporters, food additives, edible films, and coatings [2]. Additionally, the skin component of the purple-spotted bigeye was found to have 31.14% protein [7]. Fish collagen was pointed out as the most promising alternative option for high yield collagen. Due to religious restrictions, pork collagen cannot be consumed by Muslims, and its use is restricted [11].

Collagen is found in all connective tissue such as skin and bone, making it one of the most studied extracellular matrix (ECM) biomolecules [12]. Collagen has a fundamental structure in which three polypeptide (protein) chains are assembled to form a triple helix [13]. Collagen type I is the most prevalent pattern in fish, consisting of bands of α -chains (α-1 and α-2) and their dimers (ß-components) [11]. Moreover, collagen extracted from the skin of fish was frequently reported to be predominantly type I collagen, such as the skin collagen of loach (*Misgurnus anguillicaudatus*) [14], silver catfish (*Pangasius* sp.) [15], and hybrid sturgeon [16]. The intensity of protein bands containing type I collagen subunits detected in α1 was 2-fold greater than that detected in α2 [9]. Collagen’s molecular structure was recently established based on evidence from amino acid composition, analysis, electron microscopy, X-ray diffraction analysis, and physiochemical examination of solutions.

Collagen production consists primarily of pre-treatment, extraction, separation, purification, and characterization [17]. Preparation is the first step, and it varies depending on the raw materials used. Pre-treatment procedures such as washing, cleaning, and size reduction are required to reduce sample contamination [18]. The best methods for collagen preparation are acid solubilization and enzyme solubilization. The acid–collagen reaction breaks crosslinks in the collagen helix, increasing collagen extraction efficiency [2]. The acid soluble collagen (ASC) extraction of silver catfish skin using 0.5 M acetic acid yielded 4.27% [15]. With an increase in AcOH concentration from 0.1 to 2.0 M, the collagen yield increased from 0.518 to 1.581 mg/mL. In addition, according to Yu et al. [19] who optimised three critical parameters for the pepsin soluble collagen (PSC) extraction method: pepsin concentration at 1389 U/g, hydrolysis time (8.67 h) and solid–liquid (S/L) ratio of 1:57 produced a yield of 84.85%. Pepsin acts by cleaving crosslinked regions within the telopeptide without causing damage to the triple helix and increasing collagen solubility in acid [20].

Fish collagen has a wide range of applications in a variety of fields. As consumer demand for its fish increases, including from the skin of the purple-spotted bigeye snapper (*Priacanthus tayenus*), optimal exploitation of their wastes after filleting, particularly examination of its potential usage as an essential source of collagen, could be profitable. As an outcome, the goal of this study was to extract and characterise collagen of ASC and the PSC processing method from the skin of the purple-spotted bigeye, which would be of great practical value in relevant industries. As a result, information on collagen and characterisation techniques principally related to the content, properties, and structure of fish collagen are examined, as well as the possibility for usage in food applications.

## 2. Results and Discussion

### 2.1. Extraction Collagen Yield, Hydroxyproline Content, Denaturation Temperature

Table 1 shows the extraction yield, hydroxyproline content and denaturation temperature of collagen from the purple-spotted bigeye snapper skin using different acid and pepsin soluble extraction methods. On a wet weight basis, PSC (6.65%) had the highest collagen yield, followed by AAC (5.79%), CAC (4.15%), and LAC (3.19%). PSC was clearly the most efficient with the highest yield (*p* < 0.05) according to the results (6.65%). As a result, enzymatic extractions might be a more effective at extracting or solubilizing collagen from the skin than acidic extractions. Pepsin digestion typically yielded a higher yield than acid extraction collagen [21]. Recently, according to Jaziri et al. [22], extraction of lizardfish collagens from the bone using different acids (i.e., acetic acid, lactic acid, citric acid) has produced yields of extracted collagens ranging between 1.73% and 2.59%, with the highest yield (*p* < 0.05) observed in citric acid-extracted collagen, of which 2.59% has been extracted. In this study, before being digested, the fish skin was hydrolysed or treated with acetic acid, followed with pepsin enzymes to remove telopeptides. The enzyme functioned by crosslinking collagen’s non-helical ends (telopeptides) [23], deleting intermolecular bonding even when they were most stable in acidic solution without causing damage to the triple helix, and therefore increasing collagen solubility in acid medium [20].

The yield of extraction process collagen could also be improved by pre-treating connective tissues with non-specific proteolytic enzymes such as trypsin, pancreatin, ficin, bromelain, or papain [2]. This study found that 0.5 M acetic acid did not completely dissolve the fish skin. Even though acids have different chemical properties, their effects on the internal structure of collagen molecules and collagen yield vary [16]. Previously, collagen extraction in 0.5 M acetic acid was widely used. The skin of young and adult Nile perch (*Lates niloticus*) [24], as well as the skin of Baltic cod (*Gadus morhua*), are both commonly employed, with yields varying from 2 to 90% [25]. The yield of extracted collagen is also highly dependent on the animal species, age, and extraction parameters. Furthermore, collagen peptide contains amino groups in its terminal and side chains. After treating purple-spotted bigeye snapper skin with acid, the amino-group of collagen peptide combined with acid. Due to the dissociation of the ionic and hydrogen bonds in collagen, it can swell and dissolve in acid solutions. The reduced collagen yield might well be due to increased hydrophobic-hydrophobic interaction between protein chains due to increased ionic strength [26]. The shift in ionic strength would have reduced collagen release from skin tissues, and subsequent digestion with pepsin would have broken cross-linked molecules, increasing the collagen extraction rate [27]. Alkaline pre-treatment with a solution of sodium hydroxide (0.1 M), sodium chloride (NaCl), or sodium bicarbonate (NaHCO_3_) has been used to break down the non-collagenous proteins and pigments contained in skin raw materials [9]. As a consequence, a porous, decalcified raw material with a greater surface area is generated, which is completely prepared for collagen extraction. For instance, Butyl alcohol is used to remove lipids and fats from pre-treated fish skins prior to collagen extraction [28]. Furthermore, continuous stirring and temperature control will change the composition of the extracted and purified collagen.

Based on Table 1, the thermal transitions of extracted collagens from the skin of the bigeye snipper rehydrated in 0.05 M acetic acid show a maximum temperature (*T*_max_) corresponding to denaturation temperatures of acetic acid-ASC, lactic acid-ASC, citric acid-ASC and pepsin-PSC, which were 31.4, 31.7, 31.5, and 33.2 °C, respectively. The denaturation temperature of ASC was lower than that of PSC, indicating that pepsin digestion did not disrupt the structure of collagen. In PSC, the elimination of the telopeptide region explains the higher *T*_max_. The *T*_max_ of the purple-spotted bigeye collagen was lower than that of terrestrial animals such as calf skin (37 °C) [29]. It was discovered that this observation was in agreement with research conducted on the skin of the giant grouper (31.7 °C) [30]. In addition, *T*_max_ on collagen extraction is also greater in fish such as the brown-striped red snapper (*Lutjanus vitta*) (30.5 °C) [31] and bigeye snapper (31 °C) [32]. In contrast, several temperate- and cold-water fish, including deep-sea redfish (*Sebastes mentella*) (16.1 °C), Baltic cod skin (15 °C) [25], and arabesque greenling (15.5 °C) [33]. The variation in *T*_max_ between collagen from various species could be related to the amount of amino acids present, the amount of hydroxylation of proline residues present, particularly their hydroxyproline level, the temperature of the eco system and the temperature of the body [9,29]. It is possible to build hydrogen-bonded networks when the hydroxyl groups of hydroxyprolines in one strand are coupled to the upper chain amide carboxyl in another chain by the use of water molecules. Furthermore, high amino acid content is required to stabilise collagen [34]. Hydroxyproline is a valuable amino acid found in collagen that was utilised to assess the extraction process’ yield and efficiency [35]. Table 1 shows that pepsin-PSC extraction had a higher content of hydroxyproline than acidic extraction ASC of acetic acid (64.5, 0.92 mg/g sample), lactic acid (63.3, 1.13 mg/g sample), and citric acid (60.7, 1.85 mg/g sample). The extract had a higher collagen content, according to this result. After extraction, centrifugation may remove non-collagenous substances [34]. The extracted collagen had higher hydroxyproline and collagen contents when pepsin was used in the extraction process. A study by Kittiphattanabawon et al. [32] found that hydroxyproline content varies by fish species, environment, and body temperature of fish. Hydroxyproline instead supports inter-chain hydrogen bonding, which stabilises collagen’s triple helical structure.

### 2.2. Characterization of Collagen

#### 2.2.1. Ultraviolet (UV) Absorption Measurements

**Figure 1:**
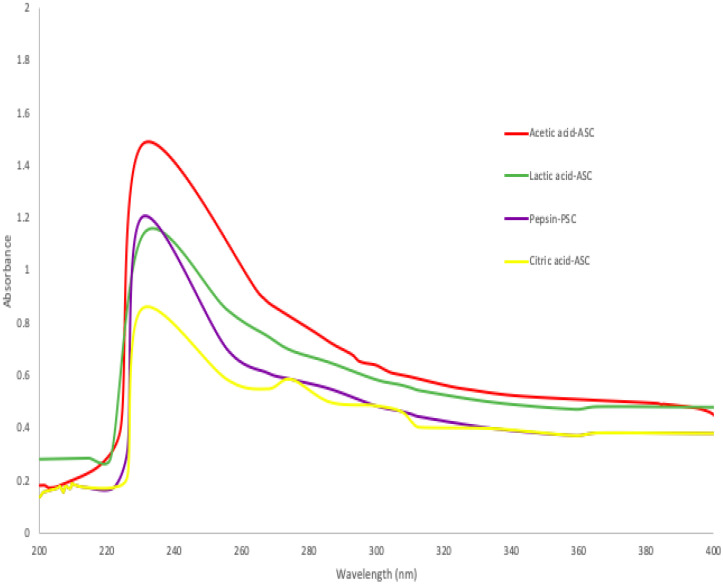
Ultraviolet spectra of collagen extracted ASC and PSC from skin of purple-spotted bigeye.

Generally, collagen and proteins have strong UV absorption due to their peptide connections and side chains. A 230 nm maximum absorbance of triple-helical collagen was due to glycine, proline and hydroxyproline [36]. According to Figure 1, maximum absorbance peaks were observed between 230 and 240 nm, when acetic acid, lactic acid, citric acid, and pepsin were used in the extraction of collagen from skin of bigeye fish. These values were comparable to those obtained from the skins of Horse Mackerels (*Magalaspis cordyla*) and Croaker (*Otolithes ruber*) [37], catla (*Catla catla*), rohu (*Labeo rohita*) [38], and scales and skin of tilapia (*Oreochromis niloticus*) [39]. Additionally, protein absorbs light most efficiently at a wavelength of 280 nm. Generally, the amino acid composition of collagen, such as tryptophan, tyrosine, phenylalanine, and other amino acids, was determined using a HPLC protocol or an amino acid analyser [16]. Additionally, amino acids such as histidine, tryptophane, phenylalanine, and tyrosine have absorption bands between 250 and 288 nm [22], whereas the collagen extracted from the skin of purple-spotted bigeyes had a smaller absorption wavelength. Furthermore, absorbance values between 200 and 220 nm were attributed to structural materials such as -COOR or -COOH [36].

#### 2.2.2. SDS-PAGE Patterns

**Figure 2:**
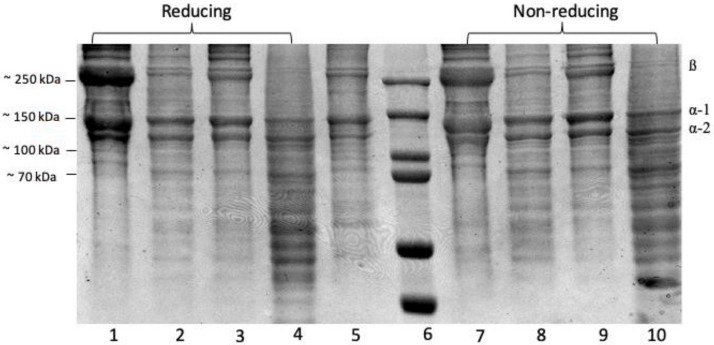
SDS-PAGE- Electrophoresis analysis of collagen extractions from the skin of purple-spotted bigeye: (reducing 1–4); Lane 1, Acetic acid; lane 2, Citric acid; lane 3, Lactic acid; lane 4, Pepsin; lane 5, type I collagen from bovine and lane 6, protein marker; (non-reducing 7–10); Lane 7, Acetic acid; lane 8, Citric acid; lane 9, Lactic acid; lane 10, Pepsin.

The SDS-PAGE profiles of the protein patterns (Figure 2) show a clear 250 kDa band linked to two other bands to form a β band, indicating the presence of α chain crosslinking linkages. Bands between 130 and 100 kDa matched the various chains—type I collagen has two α1(I) chains and one α2(I) chain [8]. All collagen samples ASC and PSC from the purple-spotted bigeye’s skin were identified as type I collagen. There were no differences in protein patterns determined under reducing and non-reducing conditions (with or without beta-mercaptoethanol) for the collagen sample, indicating that the collagen structure lacks an interchain disulphide bond. Acetic acid extraction of ASC resulted in a higher concentration of α-chains and a lower concentration of α-chains when compared to citric acid and lactic acid extractions. The purple-spotted bigeye collagen electrophoretic pattern resembles the reference type I collagen from bovine tendon. These findings corroborated those previously reported for collagens extracted from the skins of southern catfish (Silurus meridionalis Chen) [9], arabesque greenling [33], and hybrid sturgeon. Additionally, the reference collagen was placed low to the bands, indicating a decreased molecular weight, which could be due to non-collagenous proteins or collagen peptides generated during partial hydrolysis, whereas a group of small peptides that can be obtained by enzymatic action in acid at a specific incubation time [16,40]. Additionally, the molecular weights of the β-dimer band (200 kDa) and α-chains (118 kDa), and one α2 chain (106 kDa) of pepsin-solubilised collagen were slightly lower than those of acid-solubilised collagen, of which two identical α1 chains (120 kDa), and one α2 chain (110 kDa), and one β dimer band of about 203 kDa can be observed. This result demonstrated that collagen extraction with proteolytic enzymes such as pepsin was capable of cleaving and removing small segments of peptides in the telopeptide region of collagen and expressing in low molecular weight components [34]. Furthermore, the absence of telopeptides causes an amorphous arrangement of collagen molecules and a loss of the collagen fibril pattern in the reconstituted product because the C- and N-terminus telopeptides play a role in cross-linking and fibril formation [41].

#### 2.2.3. Fourier Transform Infrared (FTIR) Spectra

The infrared (IR) transmittance spectra of the collagen obtained from ASC and PSC from purple-spotted bigeye skin indicates the presence of five major amide group absorptions as determined by FTIR spectra and Table 2. Each collagen sample exhibited five major distinctive peaks, which are amide A and B bands, as well as amide I, II, and III peaks. Both the ASC and PSC spectra from the skin of bigeye snapper are similar to those of other fish species’ collagens [9,16]. The amide A is a chemical compound that has a number of functions. It is created by a free NH stretching vibration accompanied by hydrogen bonding [29]. This band was detected at 3427, 3435, 3439, and 3401 cm^−1^ for acetic acid-ASC extraction, lactic acid-ASC extraction, citric acid-ASC extraction, and pepsin-PSC extraction, respectively. These results indicate the presence of stronger hydrogen bonds in skin-PSC than in ASC, which resulted in the twisting of collagen’s triple helical structure [16]. At 2922–2931 cm^−1^, the amide B peak was discovered due to the asymmetric stretching of CH_2_ groups [23]. These bands were identified as amide B peaks in the FTIR spectra of ASC and PSC, respectively, at 2926 and 2933 cm^−1^. The amide I peak is located between 1600 and 1700 cm^−1^ and typically observed in the presence of peptides having secondary structure [24]. This frequency spectrum is created by the stretching vibration of the carbonyl group (C=O band) in the polypeptide backbone, which is responsible for the stretching vibration [9]. The collagens studied had amide I peak frequencies ranging between 1638 and 1649 cm^−1^ for ASC and PSC, respectively. The amide II is primarily associated with the in-plane bend of the N-H atom and the stretching vibration of the C-N atom. The amide II transmittance peaks of PSC were found to be lower (1555 cm^−1^) than those of acetic acid (1560 cm^−1^), implying a greater proportion of hydrogen bonds in PSC.

**Figure 3:**
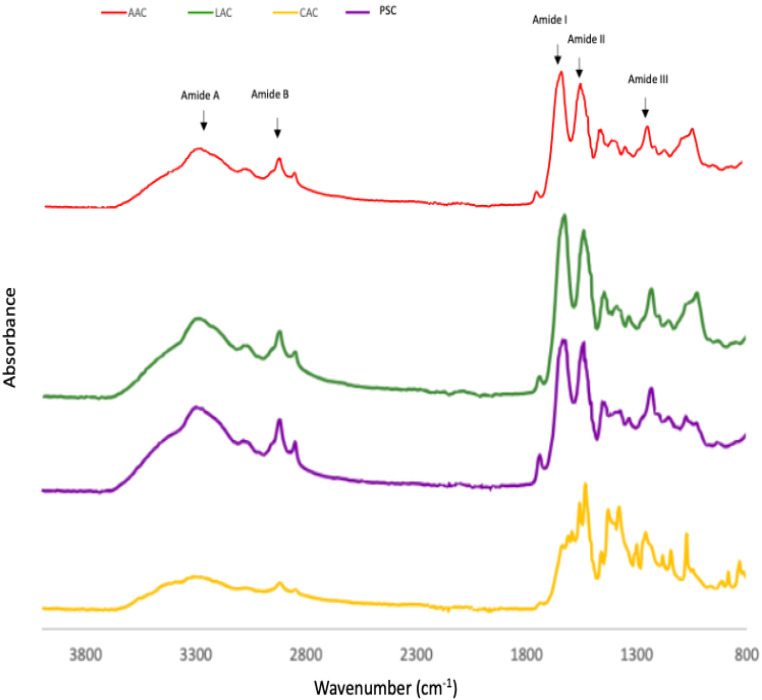
FTIR patterns of purple-spotted bigeye snapper skin collagen extracted with different acids and pepsin. AAC: Acetic acid collagen; LAC: Lactic acid collagen; CAC: Citric acid collagen; PSC: Pepsin soluble collagen.

According to Nalinanon et al. [34], pepsin was able to break the telopeptide regions’ nonhelical section, hence increasing the amount of PSC’s helical structure stabilised by the H-bond. Amide III peaks included absorption induced by CH_2_ wagging vibrations in the glycine backbone and proline sidechains, as well as C–N stretching vibrations and N–H deformation caused by amide links [20]. ASC and PSC amide III peaks were seen at 1242 cm^−1^ and 1240 cm^−1^. The amide III peak is typically located between 1220 and 1220 cm^−1^ and is caused by the NH bend in conjunction with the CN stretch, which is in charge of maintaining the helical integrity of collagen [29]. This study indicates that the collagen’s triple-helical structure retained its integrity in each sample [9]. Figure 3 demonstrates the absorption rate readings for collagen of the skin of the purple-spotted bigeye that was subjected to various acid treatments, specifically of ASC and PSC.

#### 2.2.4. Solubility of Collagen

**Figure 4:**
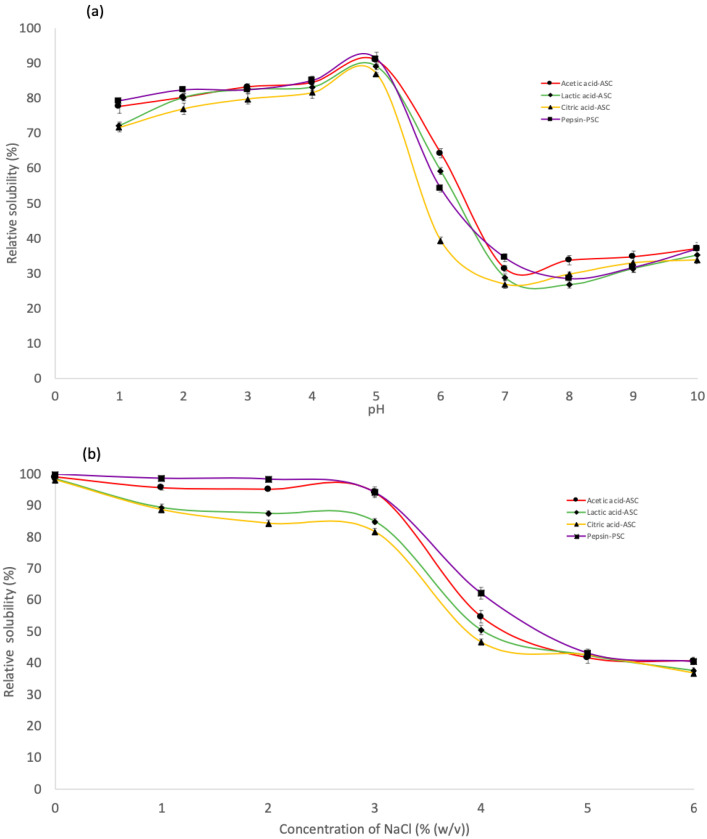
Relative solubility of the skin purple-spotted bigeye snapper collagens. (**a**) Effect of the collagen at different pH. (**b**) Effect of the collagen at different NaCl concentrations. Circle—Acetic acid collagen, AAC; Diamond—Lactic acid collagen, LAC; Triangular—Citric acid ollagen, CAC; Square—pepsin soluble collagen, PSC.

The effect of various pH values on the solubility of derived collagens is illustrated in Figure 4a. All collagen extracted by ASC and PSC were highly soluble in acetic conditions, with the maximum solubility in the pH range of 2–5. For all collagens, solubility decreased gradually from its peak value to its minimum value at pH 7. Nurkhoeriyati et al. [42] found pH 5–6 as the lowest solubility of duck protein. The influence of pH on collagen solubility is related to the protein’s isoelectric point (pI): the point at which the protein’s total charge approaches zero [35]. However, solubility continued to rise with rising pH in the alkaline pH range of 7 to 10, owing to the neutralising effect of the collagen molecules [20]. This is due to the increased hydrophobic–hydrophobic interaction between collagen molecules, resulting in a total net charge of zero, particularly at pI [23]. Collagen’s pIs should be in the neutral or alkaline pH ranges, as reflected by their lowest solubility in these pH ranges [35].

ASC and PSC solubility followed a similar pattern, with only a minor difference at various NaCl concentrations (Figure 4b). The solubility of all extraction collagen remained high in the presence of NaCl up to a concentration of 3% (*w*/*v*). Meanwhile, the solubility of collagen decreased gradually with increasing NaCl concentration and sharply decreased when the NaCl concentration was 4% (*w*/*v*), after which the solubility remained constant at a low level until 6% (*w*/*v*). The results were comparable to the solubility of collagen from barramundi skin (*Lates calcarifer*) by Jamilah et al. [35] and haruan scales (*Channa striatus*) by Pamungkas et al. [20]. A higher concentration of NaCl may result in decreased protein solubility via a “salting out” effect by increasing hydrophobic interaction between protein chains and increasing competition for water with ionic salts, resulting in protein precipitation [20,34]. Consequently, at low salt concentrations, protein solubility often increases slightly throughout the salting-in procedure [43]. Furthermore, at all NaCl concentrations tested, PSC was more soluble than ASC. According to Pamungkas et al. [20], employing pepsin in collagen extraction may promote the partial hydrolysis of high MW crosslinked molecules, leading to increased pepsin solubility [44]. Furthermore, according to Jamilah et al. [35]^,^ as the collagen molecule’s cross-linking strengthens, it might be less soluble in solvents such as salt and acid solutions. It has been claimed that the greater solubility of the later collagen was caused by the protease altering the collagen structure and shortening the collagen chain [34]. Furthermore, these findings indicate that PSC extraction is more effective in terms of the properties investigated in this study.

### 2.3. Remarks

Therefore, collagen extracted from the skin of the purple-spotted bigeye fish has the potential to contain bioactive peptides such as antimicrobial, immunomodulatory, antioxidative, and ACE-inhibiting peptides. There is a possibility that the collagen oligopeptides found in fish skin contain a high concentration of bioactive peptides [45]. As a consequence, we will examine the food, nutraceutical, and dietary supplement industries for opportunities to include collagen and peptide as bioactive ingredients. Some relevant experiment designs could be conducted, since this collagen has the potential in food application to be used as a gelling, stabilising, foaming, and emulsifying agent in food products, allowing it to be used as an active food packaging material. Currently, blending two or more biopolymers yields packaging films with better physical and mechanical properties. Chitosan, gelatin, banana starch, collagen, and alginate has been used [46,47]. Many studies have shown active packaging by incorporating plant extract, essential oils, gels, and protein isolates [48,49]. A higher concentration of polyphenols may exhibit antioxidant and antimicrobial activity and could be used as a natural food preservation additive. As a result, this collagen blend with chitosan and pomegranate peel extract would be suitable for food product protection. Collagen-chitosan blended films could be tested for antimicrobial properties against various food-borne pathogens. Furthermore, previously used fish waste collagen was successfully extracted and used to develop an active food packaging film with antibacterial properties. In addition, Nesse et al. [50] discovered that potential fish collagen contains the highest amount of essential amino acids, which paves the way for its utilisation as a dietary supplement. Despite the fact that this primarily consists of free amino acids and short-chain peptides, it is safe and effective for children who are undernourished. Due to their moisture absorption properties, collagen and its fractions have demonstrated a significant role as valuable nutritive fibres and protein sources in human diets [2]. Collagen supplements may help with the treatment of degenerative bone and joint diseases, increase lean muscle gain, shorten recovery time, fix damaged joint structure, and improve cardiovascular performance [51]. Collagen promotes natural creatine, a key amino acid in post-workout muscle growth. In the field of sports nutrition, arginine found in hydrolysed collagen promotes muscle mass and demand [52]. Furthermore, collagen-rich fish hydrolysates can be employed as food components and additives to promote emulsification, foaming, or dispersion activities [53], making them more valuable as multipurpose functional ingredients in processed foods. Collagen-containing protein hydrolysates with antioxidant activity can also be utilised to improve the shelf life of food products. Dekkers et al. [54] reported that Tilapia protein hydrolysates enhanced the stability and shelf life of carp loach. Bioactive peptides with antioxidant, anti-inflammatory, anticancer, neuroprotective, or antihypertensive activities of bigeye fish skin collagen can be used in functional foods, pharmaceutical products, and cosmetics.

## 3. Conclusions

According to this study, collagens separated from the skin of the purple-spotted bigeye by several acidic extraction ASC procedures using acid organic (i.e., acetic acid, lactic acid, and citric acid) and enzymatic extraction PSC by pepsin may have an effect on the yield and properties of collagen. In terms of ASC extraction, the maximal collagen yield in AAC extraction was significantly higher than in other acids. However, PSC was found to produce more collagen than AAC. Additionally, every collagen extracted was type I and retained its triple helix form. Moreover, the hydroxyproline concentration and thermal stability of the PSC demonstrated that it possesses superior properties to those of CAC, LAC and AAC samples of collagen obtained from purple-spotted bigeye skin.

## 4. Materials and Methods

### 4.1. Raw Materials Preparation

**Figure 5:**
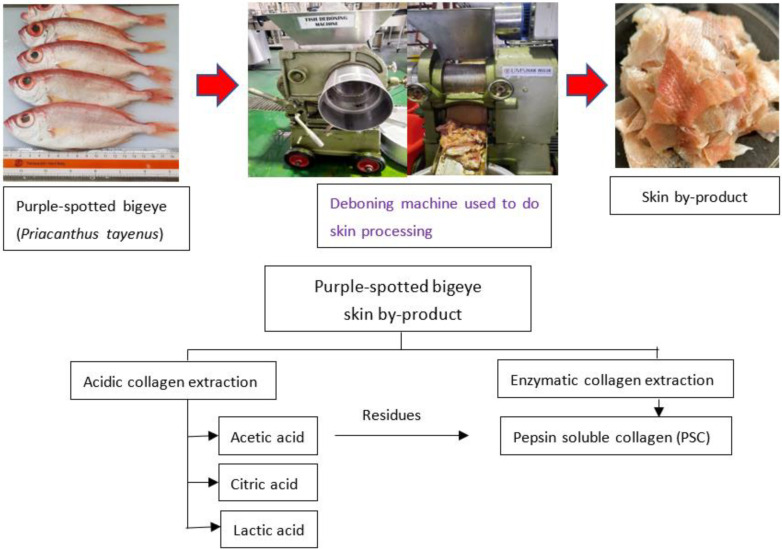
Schematic representation of the extraction processes to obtain collagen from skin purple-spotted bigeye snapper (*Priacanthus tayenus*).

The twenty kilograms of purple-spotted bigeye (*Priacanthus tayenus*) was purchased from Kota Kinabalu’s fish market. The weighted fish samples were (105.46 ± 1.05 g) and (18.67 ± 1.41 cm) in length. The skin of the bigeye snapper was removed using a mechanical deboner machine (SFD-8, 137, Ding-Han Machinery Co., Ltd.， Taipei, Taiwan) (Figure 5). The skin was collected, and the debris manually removed. The prepared skin was washed with tap water. A polyethylene bag was then placed over the skin. The skin was kept at −20 °C until used.

### 4.2. Pre-Treatment and Defatting Process

A solution of 0.1 N NaOH 1:10 (*w*/*v*) ratio was used to remove non-collagenous proteins from the prepared fish skin. The alkali solution was changed every 60 min for 6 h at 4 °C. NaOH is acceptable for pre-treating skin because it causes swelling, which improves collagen extraction by increasing the mass transfer rate in the tissue matrix [21]. The chemically altered samples were followed by solvent change every 12 h with cold water washing to neutralize to pH 7.0. Next, the skin samples were defatted in a 1:10 (*w*/*v*) butyl alcohol solution for 48 h, attempting to change the solvent every 12 h. These were then washed in cold water to neutralise the (pH 7.0).

### 4.3. Extraction of Collagen 

Collagen extraction with minimal modifications were obtained using the approach described in Wei et al. [16]. The illustration of fish collagen extraction from purple-spotted bigeye (*Priacanthus tayenus*) by-products is shown in Figure 1. Fish skin was treated for 48 h prior to acid extraction with lactic acid (LAC), acetic acid (AAC), and citric acid (CAC), at a ratio of (1:15 *w*/*v*), which were carried out at a temperature of 4 °C. Next, the mixture was filtered through two layers of cheese cloth was performed. The residue was extracted immediately using the same conditions as the initial extraction. The supernatants were combined and salted using 2.5 M NaCl in the existence of 0.05 M tris-HCl (pH 7.0). After centrifugation (Eppendorf, Centrifuge 5804, Hampton, VA, USA) at 18,000× *g* for 30 min at 4 °C, the precipitated was collected and a volume of 0.5 M acetic acid was added to dissolve the compound. For 48 h, the resultant solution was dialyzed against 0.1 M acetic acid, with six-hour solution changes and 24-h dialysis with distilled water. The dialysate that was obtained following the lyophilization using a freeze-dryer (Labconco, South Kansas City, KS, USA) and labelled as ASC. The procedure for enzymatic collagen extraction was adapted from Matmaroh et al. [55] with slight modifications. The undissolved residues from the acid extraction were suspended in 0.5 M acetic acid containing 1% (*w*/*w*) pepsin for 48 h at 4 °C with constant stirring. The collagen extraction with pepsin followed the same procedure as the ASC. The collagen extracted during the extraction method was labelled as “Pepsin-soluble collagen, PSC”. Finally, all collagen extractions were lyophilized with a freeze-dryer (Labconco, South Kansas City, KS, USA) and stored at 4 °C until further analysis. The collagen extraction yield was calculated by comparing the weight of purple-spotted bigeye skin to dry defatted skin.

### 4.4. Characterization of Collagen

#### 4.4.1. Ultraviolet (UV) Absorption Measurements

The UV absorption spectra of the purple-spotted bigeye (*Priacanthus tayenus*) collagen samples were analysed with a UV-Vis spectrophotometer (Agilent Cary 60; Agilent, Santa Clara, CA, USA) according to the procedure described by Liao et al. [56] with a few slight modifications. It was required to dissolve the collagen (10 mg) in 1 mL of 0.5 M acetic acid, and to then place the sample solution in a quartz cell. The UV spectrum was measured at wavelengths ranging from 200 to 400 nm, using a baseline of 0.5 M acetic acid as the starting point. The data were recorded straight to the load data in accordance with the wavenumber set.

#### 4.4.2. Sodium Dodecyl Sulphate-Polyacrylamide Gel Electrophoresis (SDS-PAGE)

A method of SDS-PAGE was used to identify the protein pattern described by Arumugam et al. [26] and a separating gel of 8% was created by combining 30:0.8% acrylamide: bisacrylamide, Tris-HCl (pH 8.8), 20% SDS, 10% ammonium persulphate, and TEMED. For the preparation of three sample loading dyes, 25% glycine, 20% SDS, 5% β-mercaptoethanol, and 0.1% bromophenol blue were used. The gel was stained with Coomassie Brilliant Blue R-250 staining solution after electrophoresis. The Mini-PROTEAN electrophoretic system was used to run the SDS-PAGE gel.

#### 4.4.3. Fourier Transform Infrared (FTIR) Spectra

The method of FTIR was generated and previously described by Ahmed et al. [29] with slight modification. To evaluate collagen synthesis, these data were collected from KBr-discs containing 1 mg dried hydrolysate in 100 mg potassium bromide (KBr). All essential equipment was cleaned with acetone before forming disc. A sample and KBr were pulverised and combined, then palletized to make a small thin disc. A Thermo Nicolet 380 Spectrometer was then used (Fisher Scientific Inc., Hampton, NH, USA). Spectra from wavelength 400–4000 cm^−1^ were acquired at a resolution of 2 cm^−1^ using Opus software (Fisher Scientific Inc., Hampton, NH, USA).

#### 4.4.4. Hydroxyproline Content

The hydroxyproline content of all extracted collagens was determined using a slightly modified version of the method described by Nalinanon et al. [34]. In an incubator oven (Memmert U10, Schwabach, Germany) the samples were hydrolysed with 6 M HCl for 24 h at 110 °C. The hydrolysate was clarified using activated carbon before being filtered through Whatman No. 4 filter paper. To achieve a pH of 6.0–6.5, the filtrate was neutralised with 10 M and 1 M NaOH. The neutralised sample (0.1 mL) was transferred to a test tube and isopropanol (0.2 mL) was added and thoroughly mixed; 0.1 mL of oxidant solution 7% (*w*/*v*) chlororamine T and acetate/citrate buffer, pH 6, at a ratio of 1:4 (*v*/*v*)) was added and thoroughly mixed, as well as 1.3 mL of Ehrlich’s reagent solution (mixture of solution A and solution B). The mixture was stirred and boiled in a water bath (Memmert GmbH, Schwabach, Germany) at 60 °C for 25 min before being cooled with running water for 2–3 min. Isopropanol was used to dilute the solution to 5 mL. At 558 nm, absorbance was measured in comparison to water. There was also a hydroxyproline standard solution with concentrations ranging from 10 to 60 ppm. The hydroxyproline content was expressed in mg/g of sample.

#### 4.4.5. Differential Scanning Calorimetry (DSC)

Collagen is denatured at a specific temperature, which can be determined using the DSC method developed by Seixas et al. [8] with slight modification. Rehydration of skin samples was performed using deionized distilled water or 0.05 M acetic acid at a solid/solution ratio of 1:40 (*w*/*v*). For two days, the solution was maintained at 4 °C. DSC was performed using a Model DSC 7 (Norwalk, CT, USA). Temperature calibration was performed using an Indium thermometer. The samples (5–10 mg) were weighed precisely and then sealed in aluminium pans. The samples were scanned at a rate of 1 C/min over a temperature range of 20–50 °C while being chilled using liquid nitrogen. As a reference, an empty pan was used. The area in the DSC thermogram was used to calculate total denaturation enthalpy (∆H). The thermogram was used to calculate the maximum transition temperature (*T*_max_).

#### 4.4.6. Solubility of Collagens

The solubility of collagens at various pH and NaCl concentrations was determined according to the method of Jaziri et al. [22] with slight adjustment. All collagen samples were dissolved for 12 h at 4 °C with constant stirring in 0.5 M acetic acid. Eight mL of collagen solution (3 mg/mL) was transferred to a centrifuge tube, and the pH was varied to a range of 1 to 10 using either 6 M NaOH or 6 M HCl. To produce a final volume of 10 mL, distilled water was added to the solutions. The collagen solutions were then centrifuged for 30 min at 10,000× *g* at 4 °C. To generate final NaCl concentrations of 1–6% (*w*/*v*), five mL of collagen solution (6 mg/mL) was combined with five mL of cold NaCl. Gently stirring the solutions for 60 min at 4 °C followed by centrifugation (Eppendorf, Centrifuge 5804, Hampton, VA, USA) at 10,000× g for 30 min at 4 °C. The amount of protein in the supernatant was evaluated using the Lowry method, which used bovine serum albumin (BSA) as a reference to estimate the protein content. The following Equation (1) was used to estimate the relative solubility of the compounds:

(1) $$Relative\;solubilty\;\left(\%\right)=\frac{\;Current\;concentration\;of\;protein\;}{The\;highest\;concentration\;of\;protein}\;\times \;100$$

### 4.5. Statistical Analysis

Statistical analyses were performed using SPSS Statistics 27.0. (IBM Corp., Armonk, NY, USA). The data were reported as means ± standard deviation of three independent replicates. The LSD test was used for numerous comparisons of means. Statistical significance was defined as *p* < 0.05.

## Figures and Tables

**Table 1:** Extraction yield, hydroxyproline content, denaturation temperature and enthalpy of extraction collagen, ASC and PSC from the skin of purple-spotted bigeye.

Characteristics	Acetic Acid-ASC	Lactic Acid-ASC	Citric Acid-ASC	Pepsin-PSC
Yield (% dry weight)	5.79 ± 1.53 ^c^	3.19 ± 1.34 ^a^	4.15 ± 0.93 ^b^	6.65 ± 0.98 ^d^
Hydroxyproline (mg/g sample)	64.5 ± 0.92 ^c^	63.3 ± 1.13 ^b^	60.7 ± 1.85 ^a^	66.3 ± 1.03 ^d^
Transition temperature, *T*_max_ (°C)	31.4 ± 0.53 ^a^	31.7 ± 0.32 ^a^	31.5 ± 0.71 ^a^	33.2 ± 0.62 ^b^
Enthalpy, ΔH (J/g)	0.607 ± 0.15 ^a^	0.950 ± 0.08 ^a^	0.703 ± 0.13 ^a^	1.213 ± 0.06 ^b^

Means ± SD from triplicate determinations. Different letters (a–d) in the same column indicate the significant difference (*p* < 0.05).

**Table 2:** FTIR spectra peak locations and their assignments for ASC and PSC from the skin of purple-spotted bigeye snapper.

Region	Peak Wavenumber (cm^−1^)				Assignment
	AA-ASC	LA-ASC	CA-ASC	PSC	
Amide A	3427	3435	3439	3401	NH stretch coupled with hydrogen bond
Amide B	2926	2928	2927	2933	CH2 asymmetrical stretch
Amide I	1638	1643	1642	1649	C=O stretch/hydrogen bond coupled with COO-
Amide II	1560	1563	1564	1555	NH bend coupled with CN stretch
-	1459	1460	1461	1451	CH2 bend
-	1411	1410	1410	1408	COO- symmetrical stretch
-	1325	1323	1323	1323	CH2 wagging of proline
Amide III	1242	1240	1240	1240	NH bend coupled with CN stretch
-	1084	1082	1081	1082	C=O stretch
-	620	618	618	618	Skeletal stretch

AA, Acetic acid; LA, Lactic acid; CA, Citric acid.

## Data Availability

The data presented in this study are available upon request from the corresponding author.
